# The Nedd8 Non-covalent Binding Region in the Smurf HECT Domain is Critical to its Ubiquitn Ligase Function

**DOI:** 10.1038/srep41364

**Published:** 2017-02-07

**Authors:** Shan He, Yu Cao, Ping Xie, Guanglong Dong, Lingqiang Zhang

**Affiliations:** 1 State Key Laboratory of Proteomics, Beijing Proteome Research Center, Beijing Institute of Radiation Medicine, Collaborative Innovation Center for Cancer Medicine, Beijing 100850, China; 2 Georgia Cancer Center, Augusta University, Augusta GA, USA; 3 Department of General Surgery, Chinese People’s Liberation Army General Hospital, Beijing 100853, China

## Abstract

Nedd8 is a ubiquitin-like protein that controls vital biological events through conjugation to target proteins. We previously identified the HECT-type ubiquitin ligase Smurf1 which controls diverse cellular processes is activated by Nedd8 through covalent neddylation. However, the effect of non-covalent binding to Nedd8 remains unknown. In this study, we demonstrate that both Smurf1 and its homologue Smurf2 carry a non-covalent Nedd8-binding site within its catalytic HECT domain. Structural analysis reveals that Smurf2 has Nedd8-binding sites within the small sub-domain of N-lobe and the C-lobe of HECT domain. Interestingly, the consensus Nedd8 binding sequence, L(X7)R(X5)F(X)ALQ is conserved in both Smurfs. Mutational studies reveal that all the five residues in the conserved sequence are required for binding to Nedd8. Functional studies suggest that mutations that disrupt Smurf interaction with Nedd8 reduce its neddylation and stabilize the protein. Furthermore, Nedd8 binding site in Smurf is shown to be necessary for its ubiquitin ligase activity towards the substrate and also the self-ubiquitylation. Finally, we show that Nedd8 binding to Smurf plays important roles in the regulation of cell migration and the BMP and TGFβ signaling pathways.

Nedd8 (neural precursor cell expressed developmentally down regulated protein 8) is a ubiquitin-like protein that controls vital biological processes^1^. Protein neddylation is essential in mammals^2^, plants^3^, fruit flies^4^, nematodes^4^,^5^, and budding yeast^6^. Nedd8 overexpression causes aberrant effects in cells^7^, especially in cancer cells^8^,^9^,^10^. Similar to ubiquitin, specific Nedd8 binding proteins have been identified to recognize neddylated substrates and signal downstream effects^11^,^12^,^13^,^14^,^15^. Our knowledge regarding the molecular determinants of ubiquitin-like interface and how Nedd8 binding translates into a biological function is still lacking. To date, only UBA domains of NUB1 (Nedd8 ultimate buster1), NUB1L (NUB1 long isoform)^16^,^17^,^18^ and UIM (ubiquitin-interacting motif) of UBXD7 are shown to bind to the hydrophobic patch around Ile44 amino acid of Nedd8^19^,^20^.

Smurf1 (Smad ubiquitylation regulatory factor 1) and Smurf2 (Smad ubiquitylation regulatory factor 2) belong to HECT domain ubiquitin ligases that regulate transforming growth factor (TGFβ) and BMP (bone morphogenesis protein) signalings. They play important roles in bone homeostasis mainly by degrading Smads through polyubiquitylation as well as cell motility and polarity, in part, by targeting the GTPases, RhoA and Rap1 for degradation^21^,^22^,^23^.

Although neddylation is well-known to activates the Cullin-RING type of ubiquitin E3 ligases, many non-Cullin targets of neddylation have been proposed. We recently identified Smurf1 as the first HECT ligase which can be activated by neddylation. We found that Smurf1 physically interacts with Nedd8 and Ubc12 (a E2 for neddylation), forms a Nedd8-thioester intermediate, and then catalyses its own neddylation on multiple lysine residues^24^. Lately, a non-covalent ubiquitin binding site within the HECT domain of Smurf has been characterized and proposed to play a role in facilitating polyubiquitylation and binding to ubiquitylated substrates^25^. However, the underlying mechanism of Nedd8 binding to Smurf remains unclear.

In this study, we report that similar to Smurf1, Smurf2 also binds non-covalently to Nedd8. We employed ZDOCK docking technology to map the non-covalent Nedd8 binding surface on the HECT domain of Smurf2 and identified the conserved sequence L(X7)R(X5)F(X)ALQ on the Nedd8 binding interface with Smurf that influences the autoneddylation and stabilizes Smurf protein expression. Furthermore, Nedd8 binding sequence (NBS) in Smurf was shown to be necessary for its ligase activity and for both self-ubiquitylation and ubiquitylation of substrates. Lastly, we showed that Nedd8 binding to Smurf plays important roles in cell migration and the BMP and TGFβ signaling pathway.

## Results

### Smurf2 interacts with Nedd8

**Figure 1:**
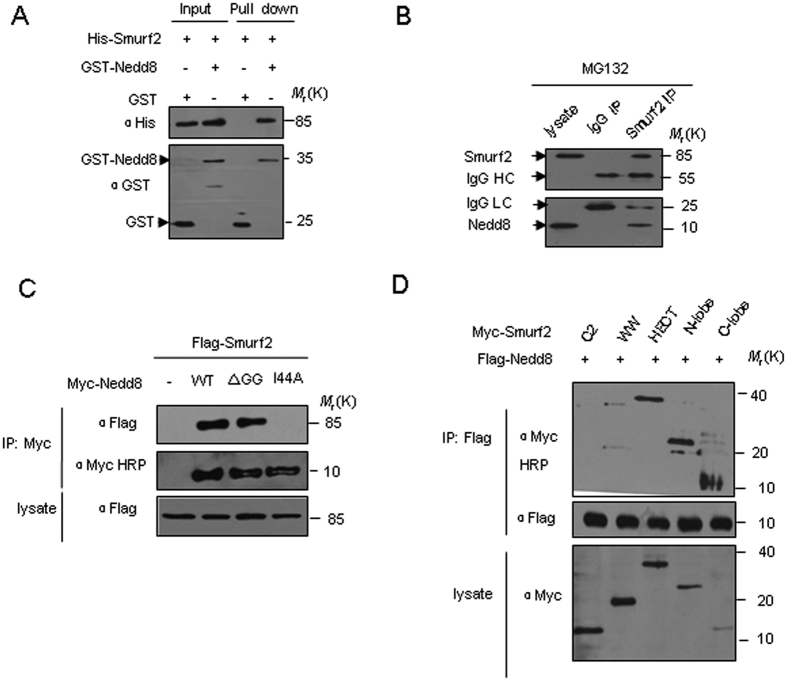
Smurf2 interacts with Nedd8 both *in vitro* and *in vivo*. (**A**) Direct interaction between Nedd8 and Smurf2 was revealed by GST pull-down assays. Both input and pull-down samples were subjected to immunoblotting with anti-His and anti-GST antibodies. Input represents 20% of that used for pull down. (**B**) Co-immunoprecipitation (Co-IP) of endogenous Nedd8 and endogenous Smurf2 from HEK293 cells. Western-blot analysis of whole-cell lysates and immunoprecipitates with Smurf2 antibody or control IgG. To avoid Smurf1 degradation, proteasome inhibitor, MG132 was added and incubated for 12 h before cells were harvested. IP, immunoprecipitate; IB, immunoblotting; IgG, immunoglobulin; IgG HC, IgG heavy chain. IgG LC, IgG light chain. (**C**) Smurf2 was co-immunoprecipitated with both wild type and ΔGG mutant forms of Nedd8 but did not interact with the mutant of I44A. HEK293T cells transfected with Myc-Nedd8 or the mutants and Flag-Smurf2 were immunoprecipitated with anti-Flag, followed by immunoblotting analysis. (**D**) Mapping of interacting regions of Smurf2 with Nedd8. Flag-Nedd8 and the indicated Smurf2 truncates were coexpressed in HEK293T cells. Cell lysates were incubated with anti-Flag antibody to precipitate Smurf2 deletion mutants. Both the lysates and the immunoprecipitates were analyzed using western blot analysis with the indicated antibodies.

To understand the mechanism of Smurf binding to Nedd8, we evaluated the non-covalent interaction between Smurf2 and Nedd8 *in vitro* and *in vivo*. Similar to Smurf1, using GST pull-down experiments, we found that Smurf2 and nedd8 can bind directly and also non-covalently, *in vitro* (Fig. 1A). In HEK293T cells, endogenous Smurf2 was detected after immunoprecipitation with an anti-Nedd8 antibody (Fig. 1B). The Smurf2-Nedd8 interaction was dependent on the Ile44-containing hydrophobic surface of Nedd8 but was indpendent on the covalent modification since the ΔGG mutant of Nedd8, which was lack of the extreme C-terminus glycine residues responsible for covalent conjugation to the substrate, retained the Smurf2-binding capacity (Fig. 1C).

We previsouly showed that the WW domain, the HECT C-lobe and the HECT N-lobe small subdomain of Smurf1 all possessed the ability to interact with Nedd8 whereas the N-lobe large subdomain of Smurf1 is responsible for ubiquitin binding^24^. Then we asked which domain of Smurf2 is responsible for Nedd8 binding. Deletion mutant analysis showed that Smurf2, like Smurf1, also contained multiple Nedd8-binding sites. The HECT N and C-lobe domain of Smurf2, but not the WW or C2 domains, possessed the ability to interact with Nedd8 (Fig. 1D). This is different from the previously identified Nedd8 binding domains, i.e. UBA domain of NUB1 and NUB1L, and UIM of UBXD7, which molecules harbor only one Nedd8 binding sequence. We also tested the other members of the Nedd4 E3 ligases and found that not only Smurf1 and Smurf2 but also NEDL1 and NEDL2 could interact with Nedd8 (Fig. S1).

### Structure docking interaction between Smurf2 and Nedd8

**Figure 2:**
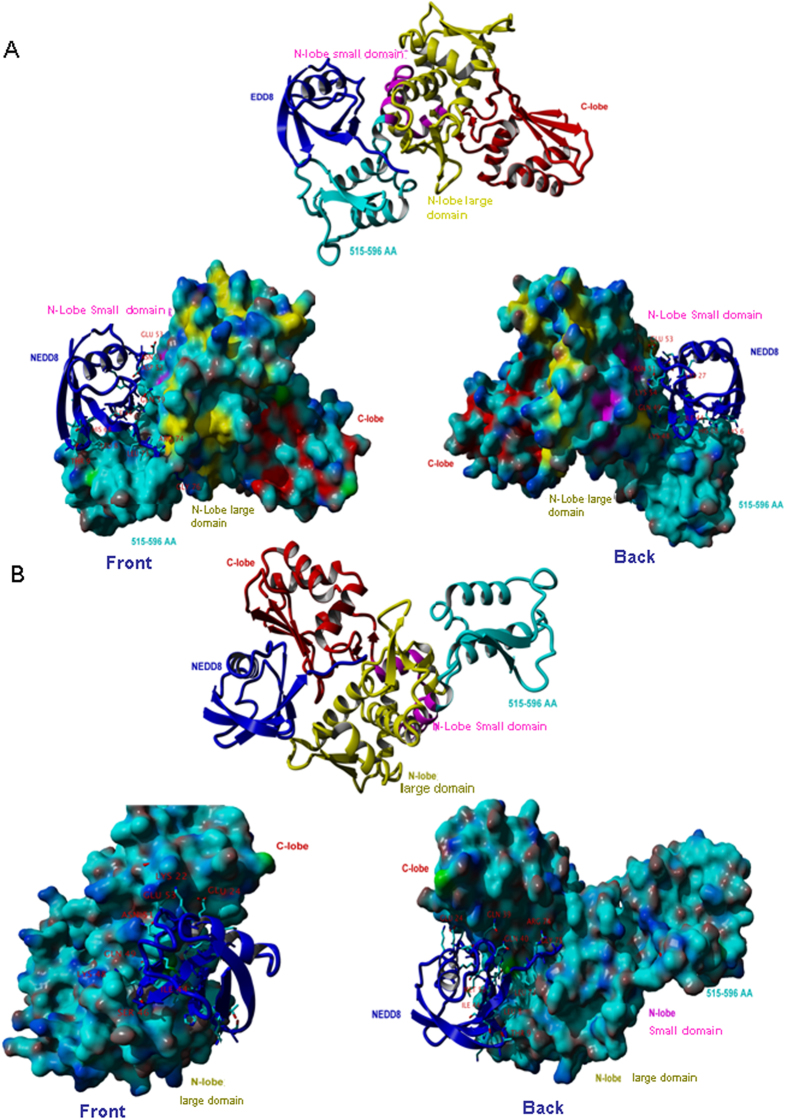
Structure docking to simulate the interaction between Smurf2 and Nedd8. ZDOCK docking procedures simulate the interaction between Smurf2 and Nedd8, set HECT domain (PDB code: 1ZVD) for the acceptor, Nedd8 (PDB code: 1XT9) is docked ligands. ZDOCK evaluated according to the geometry of the surfaces of the two molecules, hydrophobicity and polarity of the degree of matching. In total, the combination of two modes is shown in (**A,B**). Smurf2 and Nedd8 binding mode 1 is shown in (**A**) (overall view): N-lobe Small domain binding to Nedd8; different proteins and different regions are marked with different colors. The second mode is the C-lobe and details are shown in (**B**).

In order to understand the catalytic process of HECT domains, we used ZDOC docking procedures and identified the precise sites that are involved in Nedd8 interactions. By using Nedd8 (1XT9) and Smurf2-HECT (1ZVD) structures, we were able to simulate two protein interaction modes. According to a geometry analysis of the surfaces of the two molecules, matching the degree of hydrophocity and polarity, we obtained the two most optimum interaction modes between Smurf2-HECT and Nedd8 (Fig. 2A,B). The first mode is the N-lobe small subdomain of Smurf2-HECT that is most likely to bind to Nedd8 (Fig. 2A). Moreover, this is consistent with the structural chemistry of Smurf1^24^ and Smurf2. We further identified several residues which might have the potential to bind to Nedd8, such as Leu584, Leu591~Leu599, Leu601, Gln602, Asn606~Glu607 and Gln611. This showed a new, mostly acidic and hydrophobic region within the N-lobe small subdomain of the Smurf2 HECT domain apart from binding to E2 of Nedd8 and ubiquitin. The second mode of Smurf2 binding to Nedd8 we have identified is within the C-lobe domain, that is Lys644~His645, Leu672, Arg680, Phe686, Leu689, Gln690, Arg696, Pro711 and Phe717 as the potential binding sites. Although two modes of interaction were observed, only one predominant interaction occurred with a mostly hydrophobic and positively charged surface centered around Ile44 of Nedd8 (Fig. 2A,B). This is in agreement with the recently published UBA domains of NUB1, NUB1L, and UIM of UBXD7 that interact with the surface of Nedd8. However, it differs from the Smurf and Rsp5 HECT domain binding with respect to the surface centered around Arg42 and Val70 of ubiquitin^26^. Our observation also showed that in addition to the surface centered around Ile44, the Smurf2 HECT domain binding surface also includes the very C terminus of Nedd8 that is used for isopeptide linkage with a substrate lysine residue.

### Nedd8 binding sequence L(X7)R(X5)F(X)ALQ

**Figure 3:**
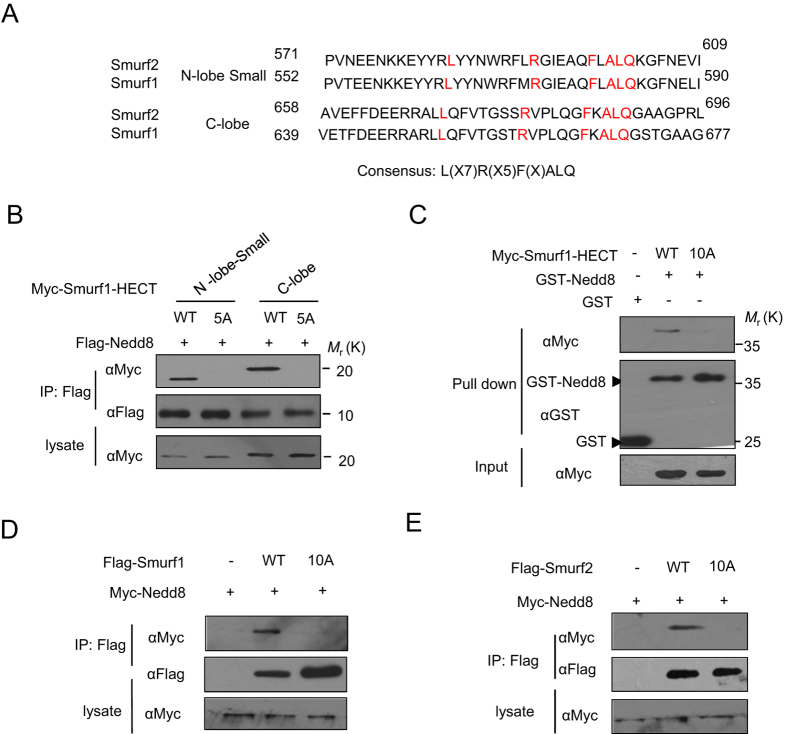
Identification of the Nedd8 binding sequence in Smurf HECT domain. (**A**) Amino acid sequence alignment of Nedd8-binding sites in N-lobe small subdomain and C-lobe domain of Smurf1 and Smurf2. The amino acid highlighted in red indicates the residues conserved in the sites for Nedd8 binding. The conserved residues are found in the sequence L(X7)R(X5)F(X)ALQ. (**B**) Co-IP assay for the interaction between Nedd8 and Smurf1 possessing L(X7)R(X5)F(X)ALQ to A(X7)A(X5)A(X)AAA substitution in the N-lobe small subdomain and C-lobe domain of Smurf1 HECT. Western-blot analysis of whole-cell lysates and immunoprecipitates with Flag antibody. (**C**) GST pull-down assay for the interaction between Nedd8 and Smurf1 possessing L(X7)R(X5)F(X)ALQ to A(X7)A(X5)A(X)AAA substitution of the whole HECT domain. Expressed Myc-tagged Smurf1-HECT (wide type or mutant) proteins in cell lysates were precipitated by GST or GST-Nedd8 from the bacterial lysates. The precipitates were analyzed by western blotting using anti-Myc antibody to detect Smurf-HECT and anti-GST antibody to detect GST or GST-Nedd8. (**D**,**E**) Co-IP assay for the interaction between Nedd8 and Smurf1/2 possessing L(X7)R(X5)F(X)ALQ to A(X7)A(X5)A(X)AAA substitution in the Smurfs. Western-blot analysis of whole-cell lysates and immunoprecipitates with Flag or Myc antibody.

We analyzed the sequences of the N-lobe small subdomain and the C-lobe domain of both Smurf1 and Smurf2; the two subdomains interact with Nedd8 at the same surface of Nedd8. All residues of the highly conserved sequences L(X7)R(X5)F(X)ALQ that we identified are in accordance with the docking results (Fig. 3A). To determine the function of the Nedd8 binding sequences of Smurf, we constructed a series of Smurf1 mutants of the key residues within the binding surface and the Leu, Arg, Phe, Leu and Gln residues were mutated together to Ala (the mutants are named 5 A and 10 A, respectively). We next evaluated whether mutation of these residues to Ala in the HECT domain of Smurfs may influence the interaction between Smurf1 and Nedd8. Our results showed that the mutations of these residues within either the N-lobe small subdomain or C-lobe domain eliminated the interaction between Smurf1 and Nedd8 (Fig. 3B). GST pull-down assays showed that mutation of all the ten residues (10 A) in the HECT domain truncate abolished the interaction between Smurf1-HECT with Nedd8 (Fig. 3C). We also tested the interaction of the mutants of the full-length Smurf1 and Smurf2. Compared to the wide-type Smurfs and Nedd8, the 10 A mutants of Smurf1 and Smurf2 lost the ability to interact with Nedd8 (Fig. 3D,E). Taken together, the L(X7)R(X5)F(X)ALQ in Smurf1 and Smurf2 proteins is a novel type of Nedd8 binding sequence.

### Nedd8 non-covalent binding region in the Smurf HECT domain contributes to its autoneddylation

**Figure 4:**
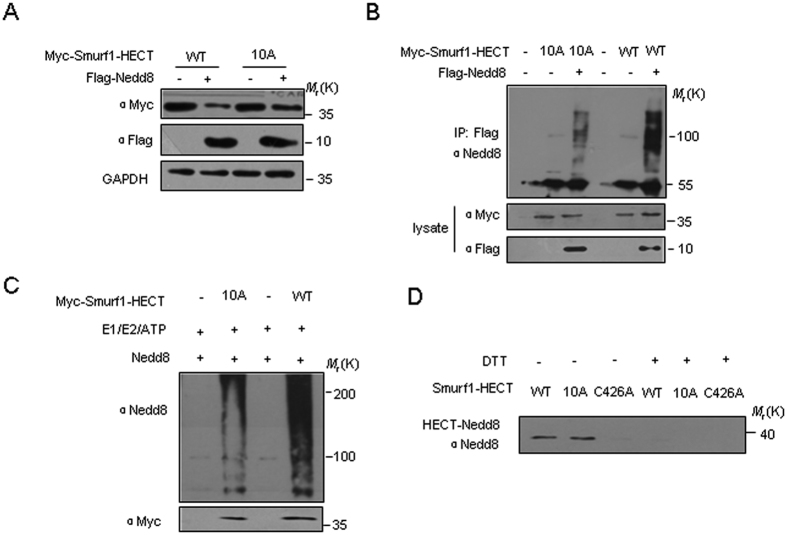
The Nedd8 binding sequence is critical for the autoneddyaltion of Smurf1. (**A**) Smurf1-HECT WT or 10 A mutant was co-expressed with Nedd8 in HEK293T cells and the expression of Smurf1 was analyzed by western blotting. (**B**) *In vivo* Smurf1 neddylationassay. HEK293T cells were transfected with Flag-Nedd8, Myc-tagged Smurf1 WT and mutants. The cells were harvested and subjected to neddylation assay 48 h after transfection. (**C**) *In vitro* autoneddylation assays were performed using purified wild-type Smurf1 or 10 A mutant together with Nedd8. Autoneddylated Smurf was analyzed by immunoblotting using anti-nedd8 antibody. (**D**) WT-Smurf1 HECT, Smurf1-HECT-10A mutant efficiently form Nedd8 thioester intermediates. Wild-type, 10 A mutant and C426A mutants were charged for 5 min at room temperature and analyzed by immunoblotting under non-reducing dithiothreitol (−DTT, lanes 1–3) or reducing conditions (+DTT, lanes 4–6) using the Nedd8 antibody.

Recently, we found that Nedd8 covalently conjugation to Smurf1 has the potential to destabilize Smurf1. We then examined whether the non-covalent binding of Nedd8 to Smurf1 had any contribution to the Nedd8 regulation on Smurf1 expression level. We found that the 10 A mutant was resistant against Nedd8 overexpression whereas wild-type Smurf1 was downregulated by Nedd8 (Fig. 4A). Neddylation assays showed that the autoneddylation of Smurf1-HECT-10A mutant was partly reduced *in vivo* and *in vitro* but not completely abolished, compared with the wild-type (Fig. 4B,C). Since the neddylation activity of the 10 A mutant was crippled, we next evaluated whether Nedd8 thioester formation was impaired. The results revealed that dithiothreitol-sensitive Nedd8 conjugation to Cys426 was comparable in both the wild-type and the 10 A mutant (Fig. 4D). Thus, although the 10 A mutation that eliminates the binding of Nedd8 interfered with neddylation, it did not affect Nedd8 charging of the catalytic cysteine to form nedd8 thioester intermediate.

### Nedd8 non-covalent binding region in the Smurf HECT domain is nessasary for its autoubiquitylation

**Figure 5:**
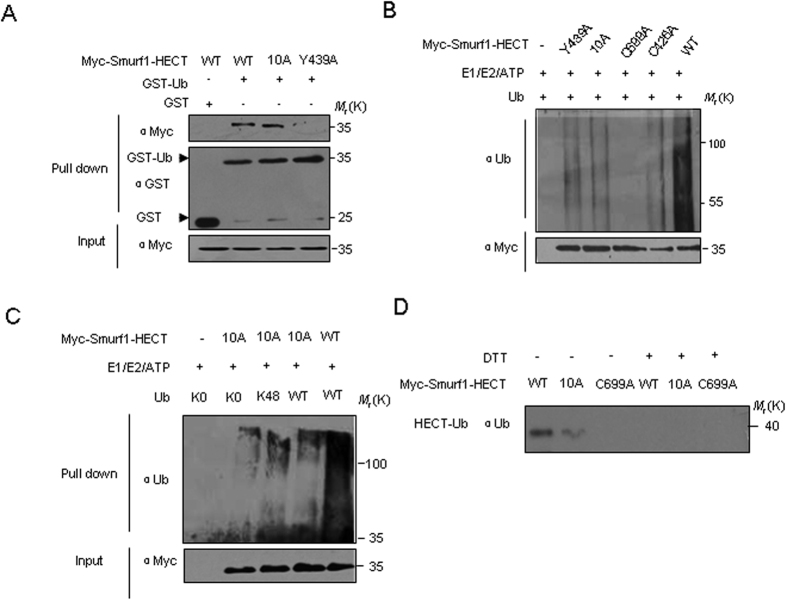
The Nedd8 binding sequence is required for autoubiquitylation of Smurf1. (**A**) Analysis of direct binding between the wide type, Y439A and 10 A mutants of Smurf1-HECT and GST-ubiquitin in GST pull-down experiments. (**B**) *In vitro* autoubiquitylation of Smurf Nedd8 binding mutant. *In vitro* autoubiquitylation assays were performed using purified Smurf1-HECT wild-type or C669A, Y439A, 10 A mutants and ubiquitin. Autoubiquitylated Smurf1 was analyzed by immunoblotting using anti-ubiquitin antibody. (**C**) *In vitro* autoubiquitylation assays were carried out using Smurf1 WT and 10 A mutants in the presence of lysine-null ubiquitin (Ubiquitin-K0) or Ubiquitin-K48 only or wide type ubiquitin and analyzed by immunoblotting with an anti-ubiquitin antibody. (**D**) Analysis of ubiquitin thioester intermediates formed by Smurf1-HECT WT, C699A and 10 A mutants with ubiquitin. The Smurf1 truncates were incubated for 10 min at room temperature and analyzed using immunoblotting under non-reducing (−DTT) or reducing conditions (+DTT) using a ubiquitin antibody.

Nedd8 is a highly conserved 81-amino acid protein that shares 60% of its identity and 80% homology with ubiquitin. A previous study has shown that the HECT domain of Smurf can interact with ubiquitin and Y439 is the key binding site of Smurf1 with ubiquitin^25^. As shown in Fig. 5A, the binding between ubiquitin and Smurf1 mutants was examined. Although Nedd8 is structural similar to ubiquitin, Smurf1-HECT-10A mutant that eliminates binding to Nedd8 did not influence the binding ability towards ubiquitin when compared with the negative control; however, as a positive control, the Y439A mutant abolished the binding to ubiquitin (Fig. 5A). This result confirms that Smurf1 binds to Nedd8 and ubiquitin through distinct regions. To examine how mutant of the Nedd8 binding sequences affected the catalytic activity of the Smurf1 HECT domain, we performed *in vitro* autoubiquitylation assays. We found that in contrast to wide-type Smurf1 that was autoubiquitylated *in vitro,* a mutation in the HECT catalytic cysteine residue (C699A) abrogated autoubiquitylation. We also examined the Y439A mutant reported to interfere with the polyubiqutylation of Smurf1 and as expected, ubiquitylation was reduced. Surprisingly, when we examined the ubiquitylated mutants that interfered with Nedd8 binding, we found that most ubiquitylation was highly reduced, similar to the Y439A mutant (Fig. 5B). This suggested that the Smurf1 10 A mutant interferes not only with neddylation but also with ubiquitylation. Further analysis showed that Smurf1-HECT-10A mutant still retained the ability to form Lys48-linkage polyubiquitin chain and also conjugate to monoubiquitin on multiple sites (Fig. 5C).

Next, we examined whether the Nedd8 non-covalent binding has any effect on the ubiquitin thioester formation of Smurf1. The results revealed that the ubiquitin thioester formation of the 10 A mutant of HECT domain is partly reduced, compared with the wild type HECT domain (Fig. 5D). These results suggest the possibility that the 10 A mutant might alter the Smurf ubiquitin E3 activity by impairing the thioester intermediate formation between ubiquitin and the cysteine residue in HECT domain.

### The mutation of Nedd8 binding sequence inhibits the ubiquitylation of substrates

**Figure 6:**
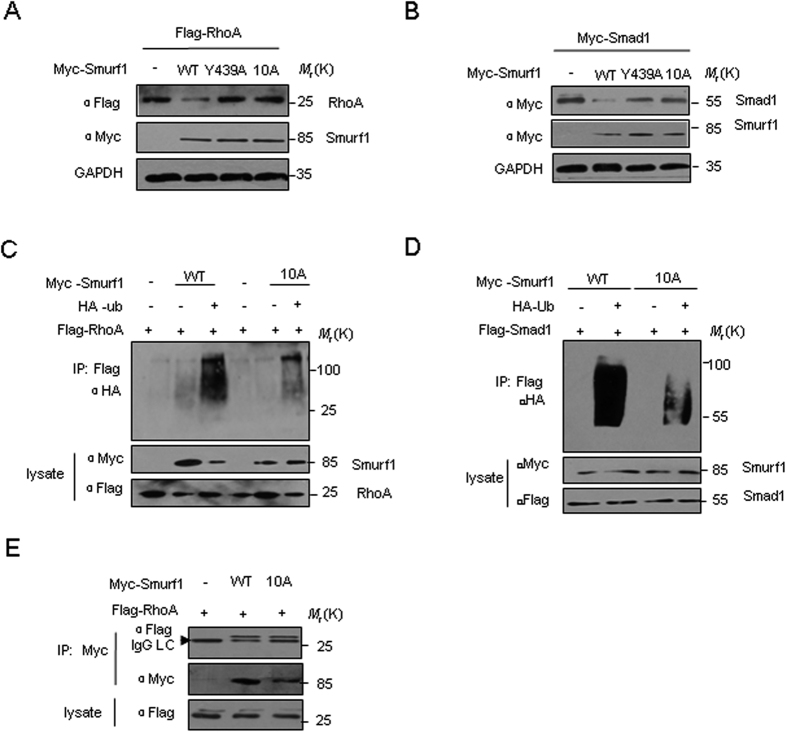
Mutation of the Nedd8 binding sequence inhibits the ubiquitylation of Smurf1 substrates. (**A,B**) The mutation in the Smurf Nedd8 binding interferes with RhoA or Smad1 targeting in cells. HEK293T cells were transiently transfected with Flag-tagged RhoA or Myc-tagged Smad1 in combination with Myc-tagged Smurf1 WT, Y439A or 10 A mutant. Steady-state levels of RhoA, Smad1 and Smurf1 in total cell lysates were determined by immunoblotting with anti-Flag and Myc antibodies. (**C,D**) Mutation in Smurf1 binding interferes with the ubiquitylation of RhoA or Smad1 in regards to targeting in cells. HEK293T cells were transiently transfected with Flag-tagged RhoA or Smad1 together with Myc-tagged Smurf1 WT or 10 A as indicated. Ubiquitylation of RhoA or Smad1 was determined by immunoprecipitation by anti-Flag followed by immunoblotting with anti-ubiquitin antibody. (**E**) Analysis of the interaction between Flag-tagged RhoA and Myc-Smurf-HECT (WT or the 10 A mutant) by Co-IP *in vivo*.

To explore the function of the Smurf Nedd8 binding sequences, we examined *in vivo,* whether the mutants of the HECT domain affected targeting of the GTPase RhoA, which is a Smurf target involved in the regulation of cell motility and polarity. We introduced the wild type Smurf1 or Y439A, 10 A mutant of Smurf1, with Flag-RhoA into the HEK293T cells. The RhoA steady-state levels were assessed through western blotting. Under these conditions, Smurf1 actively targeted RhoA for polyubiquitylation and proteasome-mediated degradation. Wild type Smurf1 strongly decreased steady-state levels of RhoA; however, both the Y439A and the 10 A mutants lost the ability to downregulate RhoA expression levels (Fig. 6A), suggesting that not only the ubiquitin-binding activity but also the Nedd8-binding activity are required for Smurf1 to efficiently degrade RhoA. Similar results were obtained for Smad1, which is also a substrate of Smurf for degradation during bone homeostasis (Fig. 6B). In order to evaluate how Nedd8 non-convalent binding to Smurf translated into a biological function, we addressed whether ubiquitylation of the Smurf substrates, such as examining whether RhoA and Smad1 were similarly affected by the 10 A mutant *in vivo*. The results showed that ubiquitylation of RhoA and Smad1 was partly inhibited by the 10 A mutants (Fig. 6C,D). Binding assays showed that RhoA binding to the wild type and the 10 A mutant HECT domains was equivalent (Fig. 6E), suggesting that the mutation might impair the ligase activity of Smurf1 rather than attenuate the Smurf1-substrate interaction.

### The Smurf-10A mutation partially inhibits the Smurf activity towards BMP and TGFβ signaling and cell migration

**Figure 7:**
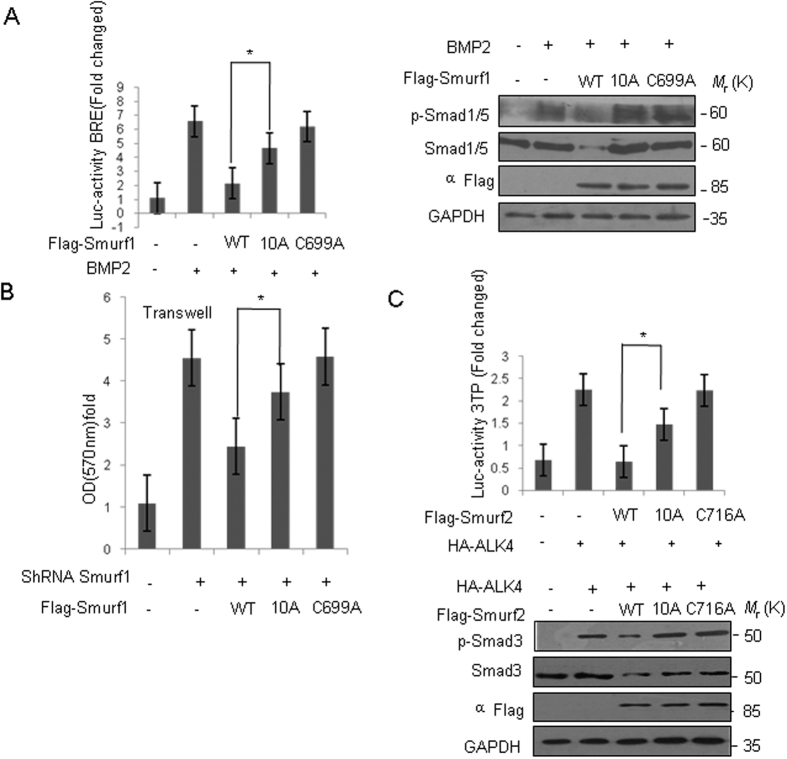
The Smurf-10A mutation partially inhibits the Smurf activity towards BMP and TGFβ signaling and cell migration. (**A**) HEK293T cells were transfected with of the indicated Smurf1 constructs and BRE-luc plasmids. Thirty-six hours after transfection, cells were treated with BMP-2 (200 ng/ml) for 12 h before BMP signaling activity was measured. Data are mean ± s.d. (n = 3). Asterisk means *P* < 0.05, student’s *t*-test. Western-blot analysis of whole-cell lysates with p-Smad1/5 or Smad1/5 antibody was also shown. (**B**) Transwell assay of the indicated HeLa cells. Data from three independent experiments were presented (mean ± S.D.) Asterisk means *P* < 0.05, student’s *t*-test. (**C**) HEK293T cells were transfected with of the indicated Smurf2 constructs and 3TP-luc plasmids. Thirty-six hours after transfection, the TGFβ signaling activity was measured. Western-blot analysis of whole-cell lysates with p-Smad3 or Smad3 antibody was also shown. Data are mean ± s.d. (n = 3). Asterisk means *P* < 0.05, student’s *t*-test.

Smurf1 negatively regulates the BMP signaling pathway and non-covalent interaction between Smurf1 and Nedd8 plays a role in the poly-ubiquitylation of Smad1. However, the precise nature of the role of this non-covalent interaction in the BMP signal pathway is unknown. To address this issue, we conducted a BMP reporter gene experiment showing that after adding BMP-2, the BMP signaling pathway became activated and overexpression of Smurf1 significantly inhibited the activity of BMP signaling. Even though Smurf1-10A mutant improved the activity of the BMP signal pathway, however it was still lower than that found within the Smurf1-C699A group (Fig. 7A), which suggests that Smurf1-10A mutant could partially inhibit the activity of Smurf1. Moreover, we also examined the expression of Smad1/5, and phosphorylated Smad1/5. BMP-2 treatment upregulated the Smad1/5 phosphorylation level and overexpression of Smurf1 WT, but not the C699A and 10 A mutants, decreased the Smad1/5 and p-Smad1/5 level (Fig. 7A).

RhoA is a member of the Rho family of small GTPases. It is mainly associated with the regulation of cytoskeleton, especially actin filament formation and actomyosin contraction. RhoA is involved in the cell growth and development. Functional incapacitation of RhoA is often considered as the cause of abnormal development of the gastrula and the loss of cell migration ability. In our preliminary experiment, we found that the Smurf-10A mutant that disrupts interaction between Smurf1 and Nedd8 could inhibit the ubiquitylation level of RhoA, but its corresponding effect on the RhoA functions remain unknown. Transwell assays revealed that cell migration abilities were increased by stable depletion of endogenous Smurf1 and then inhibited by re-introduction of Smurf1 wild-type. Compared with Smurf1 wild-type, the Smurf1-10A overexpression modestly decreased the cell migration activity whereas the catalytic inactive Smurf1-C699A mutant completely lost the inhibitory effect (Fig. 7B), suggesting that the non-covalent interaction between Smurf1 and Nedd8 is involved in the regulation of Smurf1 activity towards cell migration.

Similar to Smurf1, Smurf2 negatively regulates the TGFβ signaling pathway. The regulatory effects of Smurf2-10A on TGFβ pathway were further verified. Co-immunoprecipitation assays confirmed that Smurf2-10A could not interact with Nedd8, similar to Smurf1. Then, the possible effect of 10 A mutation on Smurf2 activity on TGFβ signalling was measured. Smurf2 overexpression inhibited ALK4-induced activation of TGFβ signalling, and this inhibition was partly reversed by Smurf2-10A, and totally reversed by Smurf2-C716A (Fig. 7C). We also tested the expression of Smad3 and phosphorylated Smad3 by overexpressed Smurf2 and its mutants (Fig. 7C). TGFβ treatment upregulated the Smad3 phosphorylation level and overexpression of Smurf2 WT, but not the C716A and 10 A mutants, decreased the Smad3 and p-Smad3 level (Fig. 7C). Taken together, these data suggest that Smurf1 and Smurf2 binding to Nedd8 plays a role in the regulation on BMP and TGFβ signaling and cell migration.

## Discussion

It is reasonable to assume that similar to ubiquitin, specific Nedd8-binding proteins recognize neddylated substrates and signal downstream effects. Indeed, several Nedd8-interacting proteins have been reported. However, in most cases we still lack knowledge in regards to the molecular determinants of the interface, its specificity over other UBLs and how Nedd8 binding translates into a biological function. Here we identified a new Nedd8 binding sequence L(X7)R(X5)F(X)ALQ through structure docking and mutant experiments. This sequence is conserved among Smurf1 and Smurf2 proteins that belong to the C2-WW-HECT ligase family. We also found that two Nedd8 binding domains, N-lobe small subdomain and C-lobe domain, had the repeat sequences L(X7)R(X5)F(X)ALQ at the HECT domain. So far as we know, only the UBA domains of yeast and human proteasomal ubiquitin-binding protein NUB1 and NUB1L have been discovered the precise Nedd8 binding sequences, that is A(X4)L(X10)L(X3)L. Notably, leucine residues are required for Nedd8 binding in Smurf1/2 and NUB1/NUB1L. And we also found that Gln and Phe residues are critical for binding and to our knowledge, this has never been shown in previous studies. This Nedd8 binding sequence may help to identify new Nedd8 interacting proteins and novel functions of neddylation in the future.

We also mapped the interaction surface on Nedd8 and showed that it is composed of a hydrophobic and positively charged surface that is centered around Ile44. Interestingly, we also observed that such interactions extended to the C terminus. This may be consistent with the notion that in the context of catalytic reactions, Nedd8 binding is manifested in the context of conjugated substrates, with the interaction surface extending to the site of conjugation. Furthermore, the surface of Nedd8 that binds the Smurf HECT domain is a common interaction surface for other Nedd8 binding domains and is also used by UBXD7. Given that Nedd8 binding to HECT domains via this surface is important for controlling neddylation, it will be interesting to discover how Nedd8-binding proteins might affect Neddylation chain addition to target substrates.

In a previous study, we showed that Smurf1 could be neddylated and activated by neddylation. It was already known that covalent modifier Nedd8 is important but how non-convalent binding influences Smurf remains unknown. In this study, we found that mutation of Nedd8 non-convalent binding reduces the neddyaltion of Smurf but does not interfere with Cys426. This suggests that Nedd8 binds to a region on the front surface of the Smurf1 HECT domain that lies separately from the conserved active site cysteine residue in the modeled structure.

Recent studies have shown that the HECT domain of Smurf has the ability to non-covalently bind to ubiquitin. The surface residues Tyr439/Tyr459 had modest effects on binding at the N-lobe large subdomain of the HECT domain. Although Nedd8 is similar to ubiquitin, our study found that the N-lobe small subdomain that binds to E2 and the C-lobe domain that has the active Cys residue could directly bind to Nedd8. The positioning of the Nedd8 surface binding differs from ubiquitin and therefore is capable of contributing to the catalytic mechanism of ubiquitin conjugation to HECT substrates. When we analyzed the activity of these Smurf HECT mutants,we observed that the mutant of the Nedd8 binding sequence L(X7)R(X5)F(X)ALQ strongly suppressed auto-polyubiquitylation as well as polyubiquitylation of the substrates RhoA and Smad1. This correlated with the inability of the 10 A mutants of Smurf1 to catalyze efficient degradation of RhoA and Smad1 in cells. Furthermore, we performed *in vitro* autoubiquitylation assays using the 10 A mutant of Smurf1 and found that the ubiquitylation of 10 A mutant was reduced compared to the wild-type Smurf1. These observations are consistent with a model in which the C-lobe binds to Nedd8 in an orientation that may change Smurf E3 activity. Since the mutant residues of C-lobe that eliminate the binding to Nedd8 are also located close to the cysteine activity sites, they may change the structure of the C-lobe domain and E3 activity by preventing ubiquitin thioester formation with Smurf1 Cys699 or Smurf2 Cys716. This explains the importance of Nedd8 non-covalent binding to Smurf and shows that Nedd8 binding to Smurf is necessary for the activity of Smurf. The finding that mutations that inactivate the Nedd8-binding site have a more pronounced effect on the Smad1 and RhoA polyubiquitylation also supports this model.

Studies on Smurf ubiquitin binding mutants led to the conclusion that the region functions predominantly in the context of polyubiquitin. Our data clearly show that the mutation of the Smurf Nedd8 binding blocks polyubiquitylation as well. Together, these studies suggest that noncovalent Nedd8 binding serves to control Smurf E3 activities through ubiquitin thioester, and also to reduce the ubiquitylation of substrates. Since ubiquitin binding mutants act to stabilize the binding of ubiquitin-conjugated substrates, we believe that Nedd8 binding is necessary for ubiquitylation and ubiquitylated substrates. It will be interesting to define how Nedd8 proteins might affect target substrates in specific cellular contexts and find their biological function in bone formation and cell migration.

## Methods

### Antibodies

All antibodies were purchased as follows: anti-Smurf1 (ab117552, Abcam), anti-Smurf2 (sc-25511, Santa Cruz), anti-p-Smad1/5 (cst9246S, Cell Signaling Technology), anti-Smad1/5 (sc6031, Sant Cruz), anti-p-Smad3 (cst9520S, Cell Signaling Technology), anti-Smad3 (cst9523S, Cell Signaling Technology), anti-ubiquitin (sc-166553, Santa Cruz), anti-Nedd8 (ALX-210-194-R200, Alexis Biochemicals), anti-Myc, anti-GST, anti-His, anti-GAPDH, anti-HA (MBL), anti-Flag (Sigma-Aldrih), and mouse/rabbit IgG (Santa Cruz).

### Plasmids and strains

Myc-Smurf1, His-Smurf2, Flag-RhoA, and Myc-Smad1 fragments have been previously described^24^. Myc-Smurf1-HECT and Myc-Smurf1/2 mutants were generated by site-directed mutagenesis at sites indicated in the text by using a point mutation kit (Agilent). Myc-Smurf1-HECT-N-lobe Small-5A (502aa-605aa, Leu564 → Ala, Arg572 → Ala,

Phe578 → Ala, Leu581 → Ala, Gln582 → Ala); Myc-Smurf1-HECT-C-lobe-5A (609aa-731aa, Leu652 → Ala, Arg660 → Ala, Phe666 → Ala, Leu669 → Ala, Gln670 → Ala); Myc-Smurf1-10A or Myc-Smurf1-HECT-10A (Leu564 → Ala, Arg572 → Ala, Phe578 → Ala, Leu581 → Ala, Gln582 → Ala, Leu652 → Ala, Arg660 → Ala, Phe666 → Ala, Leu669 → Ala, Gln670 → Ala);

Flag-Smurf2-10A (Leu583 → Ala, Arg601 → Ala, Phe607 → Ala, Leu610 → Ala, Gln611 → Ala, Leu671 → Ala, Arg679 → Ala, Phe685 → Ala, Leu688 → Ala, Gln689 → Ala). All Smurf2 fragments were cloned into pCMV-Myc vector to generate Myc-epitope tagged constructs: C2, residues 1–98; WW, residues 157–330; HECT, residues 414–748; N-lobe, residues 369–624; C-lobe, residues 628–741.

### Protein interaction docking

The structures of Smurf2-HECT and Nedd8 were obtained from the Protein Data Bank (PDB). PDB IDs are: 1ZVD (Smurf2-HECT) and 1XT9 (Nedd8). The ZDOCK docking program is adopted to take protein-protein interactions. HECT structural domain (PDB code: 1ZVD) is set as the docking receptor, and Nedd8 (PDB code: 1XT9) is set as the docking ligand. ZDOCK conducts assessments according to the geometry, hydrophobicity and the degree of polarity matching degree of two molecular surfaces.

### Co-immunoprecipitation assay

Transfection was performed using Lipofectamine 2000 (Invitrogen). Cells were harvested 24–48 h after transfection and lysated in EBC lysis buffer (0.5% NP-40, β-mercaptoethanol supplemented 50 mM Tris, pH 7.6, 120 mM NaCl, 1 mM EDTA, 50 mM NaF) and with protease inhibitor cocktail (Roche). For immunoprecipitation, 800 mg lysates were incubated with the appropriate antibody for 3 h at 4 °C followed by incubation for 1 h with Protein A/G sepharose beads (GE Healthcare). The resulting immunoprecipitates were washed at least three times in NETN lysis buffer (150 mM NaCl, 1 mM EDTA, 50 mM Tris–HCl, pH 7.8, 1% NP-40, 1 mM phenylmethylsulfonyl fluoride, 0.5 μg ml^−1^ leupeptin and 0.5 μg ml^−1^ pepstin) before being resolved by SDS-PAGE and immunoblotted with the indicated antibodies.

### GST pull-down assay

The Nedd8 sequence was inserted into the pGEX-4T-1 vector (Amersham Biosciences). Smurf2 was inserted into the pET-28a vector (Novagen). To detect direct binding, bacteria-expressed GST-tagged proteins were immobilized on glutathione Sepharose 4B beads (Amersham Biosciences) and then incubated with His-tagged proteins for 8 h under rotation. Beads were washed with GST-binding buffer (100 mM NaCl, 50 mM NaF, 2 mM EDTA, 1% NP-40 and protease inhibitor mixture) and proteins were eluted, followed by immunoblotting.

### *In vivo* modification assays

To prepare cell lysates, cells were solubilized in modified lysis buffer (50 mM Tris, pH 7.4, 150 mM NaCl, 10% glycerol, 1 mM EDTA, 1 mM EGTA, 1% SDS, 1 mM Na_3_VO_4_, 1 mM DTT and 10 mM NaF) supplemented with a protease inhibitor cocktail. The cell lysate was incubated for 10 or 30 min. The lysate was then diluted 10 times with modified lysis buffer without SDS. The lysate was incubated with the indicated antibody for 3 h at 4 °C. Protein A/G-plus Agarose (Santa Cruz) was added, and the lysate was rotated gently for 8 h at 4 °C. The immnoprecipitates were washed at least three times in wash buffer (50 mM Tris, pH 7.4, 150 mM NaCl, 10% glycerol, 1 mM EDTA,1 mM EGTA, 0.1% SDS, 1 mM DTT and 10 mM NaF). Proteins were recovered by boiling the beads in 2 × SDS sample buffer and analyzed by western blot.

### *In vitro* modification assay

*In vitro* ubiquitylation and neddylation were performed using recombinant purified enzymes. His-Smurf1 was expressed in *Escherichia coli* BL21 (DE3). For the ubiquitylation assay, 0.5 μg of His-Smurf1 or Myc-Smurf1-HECT and all Myc-Smurf1-HECT mutants that purified from the cell lysates were incubated with 0.1 μg of E1, 0.3 μg of E2 (UbcH5c or UbcH7) and 10 μg of HA–ubiquitin or ubiquitin (all from Boston Biochem) in 20 μl of ubiquitylation assay buffer (50 mM Tris–HCl, pH 8.0, 0.5 mM NaCl, 1 mM DTT, 5 mM MgCl_2_ and 2 mM ATP). For the neddylation assay, Myc-Smurf1-HECT and all mutants of Myc-Smurf1-HECT purified from the cell lysates were incubated with 2 μg of Nedd8, 0.01 μg of E1 (APPBP1-UBA3) and 0.2 μg of E2 (Ubc12) in a total reaction volume of 20 μl (40 mM Tris–HCl, pH 7.4, 5 mM MgCl_2_, 2 mM ATP and 2 mM DTT). Samples were incubated at 30 °C for 1 h and reactions were terminated with 2 × SDS PAGE.

### *In vitro* thioester bond assay

All *in vitro* Nedd8 thioester and ubiquitin thioester assays were carried out in 15 μl reaction buffer (50 mM Tris, pH 7.5, 10 mM MgCl_2_) and supplemented with 2 mM ATP; 5 × 15 μl reactions where incubation times, protein concentrations or SDS-PAGE buffer (with or without 100 mM DTT) varied using E1 (APPBP1-UBA3 or Ube1) and E2 (Ubc12 or UbcH5c), Nedd8 or ubiquitin (all from Boston Biochem), and 1 mM His-Smurf1 or the mutants. Reactions were incubated at room temperature for 5 min (Nedd8 thioester) or 10 min (ubiquitin thioester assays) and stopped with E1 stop buffer. Nedd8 thioester or ubiquitin thioester were detected by immunoblotting with Nedd8 or ubiquitin antibody.

### Cell migration assay

Cell migration assays were performed with the CHEMICON Cell Invasion Assay Kit (ECM550). First, put the small chamber into the 24-well plate. In the upper chamber, put 300 μl preheated serum-free medium. Incubate at room temperature for 30 min. Then remove the medium. Digestive cells: Use serum-free medium to resuspend cells at a density of 1 × 10^5^. Put 200 μl cell suspension into the Transwell chamber. The stable transfectant depleted of endogenous Smurf1 with shRNA or the control transfectant cells were used in the assays. In the lower chamber, add 500 μl serum medium. After 24 h, add lipofectamine 2000 to transfect Flag-Smurf1 and its C699A and 10 A mutants. After 24 h, use a cotton swab to gently wipe out the cells in the upper chamber. In every well, add 500 μl staining solution to the small chamber. After 20 min, rinse with clean water and dry. Use 100 μl acetic acid for bleaching and add to the 96-well plate. Use a microplate reader to measure the OD value at 570 nm.

### Reporter gene assay

The luciferase reporter assays were performed as we described previously^24^. Luciferase activity was measured with the Dual Luciferase Assay System (Promega) in accordance with the manufacturer’s protocol.

### Statistical analysis

Statistical evaluation was carried out using a Student’s *t*-test.

## Additional Information

**How to cite this article:** He, S. *et al*. The Nedd8 Non-covalent Binding Region in the Smurf HECT Domain is Critical to its Ubiquitn Ligase Function. *Sci. Rep.* **7**, 41364; doi: 10.1038/srep41364 (2017).

**Publisher's note:** Springer Nature remains neutral with regard to jurisdictional claims in published maps and institutional affiliations.

## Supplementary Material

Supplementary Information
