# Supplementary material for: The Nedd8 Non-covalent Binding Region in the Smurf HECT Domain is Critical to its Ubiquitn Ligase Function
Source: Sci Rep. 2017 Feb 7;7:41364. doi: 10.1038/srep41364 (PMC5294409; doi:10.1038/srep41364)
Supplement: Supplementary Information [file srep41364-s1.pdf]

Supplementary Information

The Nedd8 Non-covalent Binding Region in the Smurf HECT Domain is Critical to its Ubiquitn Ligase Function

Shan He[1], Yu Cao[1, 2], Ping Xie[1], Guanglong Dong[3*], Lingqiang Zhang[1*]

1 State Key Laboratory of Proteomics, Beijing Proteome Research Center, Beijing Institute of Radiation Medicine, Collaborative Innovation Center for Cancer

Medicine, Beijing 100850, China.

2 Georgia Cancer Center, Augusta University, Augusta GA, USA.

3 Department of General Surgery, Chinese People's Liberation Army General Hospital, Beijing 100853, China.

[*]Co-corresponding authors: Lingqiang Zhang, Email: zhanglq@nic.bmi.ac.cn or Guanglong Dong, Email: gldong301@163.com

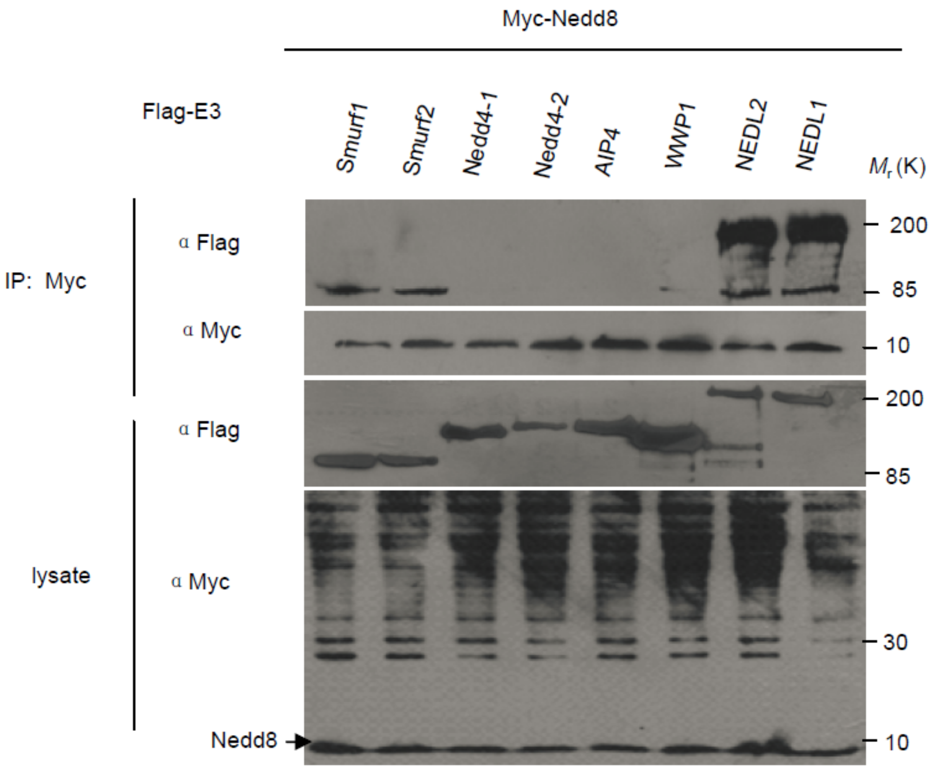

Figure S1. The interaction of Nedd4 familiy members and Nedd8 *in vivo*.

Interaction analysis of Nedd4 family E3s with Nedd8 by Co-IP assays in HEK293T cells.
